# Ovariectomy exacerbates the disturbance of excitation- inhibition balance in the brain of APP/PS-1/tau mice

**DOI:** 10.3389/fnmol.2024.1391082

**Published:** 2024-08-26

**Authors:** Fuwang Liu, Yanman Liu, Xuri Shen, Jiarui Du, Hanting Zhang, Xueqin Hou

**Affiliations:** ^1^School of Pharmaceutical Sciences and Institute of Materia Medica, Shandong First Medical University and Shandong Academy of Medical Sciences, Jinan, Shandong, China; ^2^Department of Pharmacology, School of Pharmacy, Qingdao University, Qingdao, Shandong, China

**Keywords:** disturbance of excitation-inhibition balance, Alzheimer’s disease, ovariectomy, estrogen-estrogen receptor system, APP/PS-1/tau mouse model

## Abstract

**Introduction:**

The prevalence of Alzheimer’s disease (AD) is significantly gender-differentiated, with the number of female AD patients far exceeding that of males, accounting for two-thirds of the total prevalence. Although postmenopausal AD mice have been shown to have more prominent pathologic features and memory impairments than normal AD mice, the relevant molecular mechanisms leading to these outcomes have not been well elucidated. In the present study, we used the disturbance of excitation-inhibition balance in the postmenopausal brain as an entry point to explore the link between estrogen deficiency, disorders of the glutamatergic-GABAergic nervous system, and memory impairment.

**Methods:**

Wild-type (WT) mice and APP/PS1/tau (3 × Tg-AD) mice (10 months old) were randomly divided into four groups: WT+Sham group, WT+OVX group, 3 × Tg-AD+Sham group and 3 × Tg-AD+OVX group. Ovariectomy (OVX) was performed in the WT+OVX group and the 3 × Tg-AD+OVX group, and sham surgery was performed in the WT+Sham group and the 3 × Tg-AD+Sham group. The learning and memory ability and the anxiety and depression-like behavior changes of mice were evaluated by behavioral experiments, and the association between estrogen-estrogen receptors pathway and glutamatergic/GABAergic nervous system and female AD was evaluated by neurochemical experiments.

**Results:**

In WT and 3 × Tg-AD mice, OVX resulted in impaired learning and memory abilities and anxiety and depression-like behaviors; reduced estrogen levels and downregulated the expression of estrogen receptors; upregulated the expression of amyloid-β, amyloid precursor protein, presenilin 1, and p-tau; upregulated the expression of Bcl-2-associated X protein and downregulated the expression of B-cell lymphoma-2, promoting cell apoptosis; reduced the number of neuronal dendrites and downregulated the expression of postsynaptic density protein-95; more importantly, OVX increased brain glutamate levels but downregulated the expression of N-methyl-D-aspartate receptor-2B, excitatory amino acid transporter 1, excitatory amino acid transporter 2, γ-aminobutyric acid receptor-A and γ-aminobutyric acid receptor-B.

**Conclusion:**

Our results suggested that OVX-induced estrogen-estrogen receptors pathway disruption caused learning and memory impairment and anxiety and depression-like behaviors, upregulated the expression of AD pathological markers, promoted apoptosis, destroyed neuronal structure, and most importantly, caused glutamatergic/GABAergic nervous system disorders.

## 1 Introduction

With the continuous development and advancement of modern medicine, the average life expectancy of human beings is increasing. Consequently, the incidence of geriatric diseases is increasing year by year. China is one of the most populous countries in the world and also one of the countries with the most serious aging trend, therefore, it is also facing the problem of the increasing incidence of old age diseases (especially dementia) year by year. A study on the prevalence of dementia in Chinese people over 60 years old found that the prevalence of dementia among Chinese people is as high as 6%, with Alzheimer’s disease (AD) accounting for the highest proportion of 3.9% (about 9,8 million people) (Jia et al., 2020).

AD is a neurodegenerative disorder characterized by memory amnesia in situational memory, and its most prominent pathological features are amyloid-β (Aβ) deposition, hyperphosphorylation of microtubule tau proteins, and massive loss of neurons (Villain and Dubois, 2019; Twarowski and Herbet, 2023). The onset of AD is multifactorial, including race, age, and lifestyle, gender also has a huge impact on the development of the disease (Salwierz et al., 2022; Hogervorst et al., 2023). Women are at a higher risk of developing AD compared to men (Geraets and Leist, 2023), which may be related to the dramatic decline in estrogen levels during menopause. Estrogen has been shown to have neuroprotective effects, and estrogen regulates inflammatory responses, oxidative stress, and abnormal mitochondrial function through binding to estrogen receptors (Uddin et al., 2020; Mishra et al., 2023). More importantly, normal expression of estrogen and estrogen receptors maintains the excitation-inhibition balance in the brain and thus ensures the integrity of information transmission between neurons (Blurton-Jones and Tuszynski, 2006; Karki et al., 2014; Lan et al., 2014), which is essential for memory transmission, storage and retrieval.

Disturbances in the excitation-inhibition balance have been observed in the brains of AD patients (Saba et al., 2017; Jakaria et al., 2018; Xu et al., 2020), and such disturbances consist of two main aspects: dysfunction of glutamatergic neurons and dysfunction of γ-aminobutyric acid (GABA) neurons. Glutamate (Glu) is the main excitatory neurotransmitter in the brain, and under normal physiological conditions it initiates rapid signaling by binding to Glu receptors on the postsynaptic membrane, which is subsequently taken up by excitatory amino acid transporters (EAATs) on surrounding astrocytes (Pajarillo et al., 2019), However, the metabolic collaboration between glutamatergic neurons and astrocytes in the brains of AD patients is disrupted (Andersen et al., 2021), and this may be related to a decline in estrogen, as low levels of estrogen have been found in rodent studies to lead to synaptic loss (Woolley and McEwen, 1994), in addition, increased Glu levels may have a more severe effect on women with AD, because they have lower Glu receptor expression than men at the same point in whom AD progresses, which may exacerbate the degree of synaptic loss (Wickens et al., 2018). GABAergic neurons, as the most representative inhibitory neurons, are not only involved in the occurrence and development of depression, but also play an important role in AD (Garcia-Alloza et al., 2006). GABAergic neuronal metabolism disorders were observed in the brains of AD patients (Jo et al., 2014), and a comparative study of GABA and sex noted that female AD mice had much more GABA in their brains than males (Roy et al., 2018), Interestingly, neurons that are directly targeted by estrogen in the hippocampus of the brain are also GABAergic neurons (Rai and Jeswar, 2012). Therefore, we believe that such a high incidence of postmenopausal AD may be related to estrogen-estrogen receptor-regulated glutamatergic/GABAergic neurological disorders.

Therefore, we constructed a mouse model of menopause by OVX to investigate the effects of estrogen deficiency on estrogen receptors, neurons and excitation-inhibition balance in the brain. We also elucidated the correlation between the estrogen- estrogen receptors pathway and the disturbance of excitation-inhibition balance in the brain, with the aim of providing a reference for the treatment of AD patients and the development of AD drugs in the future.

## 2 Materials and methods

### 2.1 Animals

We selected two types of 10-month-old female mice: wild-type (WT) mice and triple-transgenic (3 × Tg-AD) mice. WT mice were C57BL6/129SvJ mice and purchased from Pengyue Laboratory Animal Breeding Co (Jinan, China). 3 × Tg-AD mice carrying three mutated genes APP_swe_, PS1_M146V_ and tau_P301L_ were purchased from Jackson Laboratory (Bar Harbor, ME, USA). All mice were housed in polypropylene cages (3–4 mice per cage) in an SPF-grade animal house at the Shandong First Medical University. The animal house was kept under 12 h of light (8:00 a.m. to 8:00 p.m.), and the temperature and relative humidity of the animal house were maintained at 25°C ± 2°C and 40–50%. All mice were allowed free access to food and water. All subsequent animal experiments were conducted in accordance with the requirements of the Ethics Committee for Laboratory Animals of Shandong First Medical University (NO. 202103030154).

### 2.2 Reagents and antibodies

Sodium pentobarbital was purchased from Sigma-Aldrich (St. Louis, MO, USA). BCA protein quantitation kit (PC0020), RIPA buffer (R0010), Tris-buffered saline (TBS, T1083), Tween-20 (T8220), phosphate-buffered solution (PBS, P1020), protease (PMSF, P0100), and phosphatase inhibitor (P1260) were obtained from Solarbio (Shanghai, China). 4% neutral paraformaldehyde (G1101), electron microscope fixative (G1102), Nissl stain solution (G1036), Golgi fixative (G1069-15ML), and thioflavine S (S19293) were obtained from Servicebio (Wuhan, China). Antibodies against the following proteins were purchased from Abcam (Cambridge, MA, USA): α-amino-3-hydroxy-5-methyl-4-isoxazole-propionic acid receptor (AMPAR, ab183797), N-methyl-D-aspartate receptor-2A (NMDAR2A, ab124913), N-methyl-D-aspartate receptor-2B (NMDAR2B, ab254356), excitatory amino acid transporter 1 (EAAT1, ab181036), excitatory amino acid transporter 2 (EAAT2, ab205248), γ-aminobutyric acid receptor-A (GABAA, ab300069), γ-aminobutyric acid receptor-B (GABAB, ab238130), presenilin 1 (PS1, ab76083), B-cell lymphoma-2 (Bcl-2, ab182858), Bcl-2-associated X protein (Bax, ab32503), postsynaptic density protein-95 (PSD95, ab192757), estrogen receptor-α (ER-α, ab32063) and estrogen receptor-β (ER-β, ab187291). Anti-β-actin antibody (TA-09) and the horseradish peroxidase-conjugated secondary antibodies, including goat anti-mouse (ZB-2305) and goat anti-rabbit (ZB-2301), were purchased from Zhongshan Golden Bridge (Beijing, China). Enhanced chemiluminescence detection kits (BL520B) were purchased from Bioship (Hefei, China). Enzyme-linked immunosorbent assay kits were purchased from Shanghai Enzyme-linked Biotechnology Co. (Shanghai, China) to analyze estradiol (E2, ml063198), amyloid precursor protein (APP, ml063243), tau (ml037981), p-tau (ml037636). Glu (BPE20294) and GABA (BPE20434) were purchased from Shanghai lengton Biotechnology Co. (Shanghai, China).

### 2.3 Drugs and treatments

**FIGURE 1:**
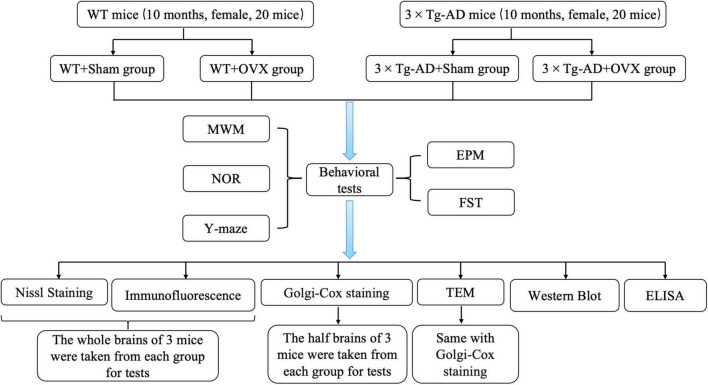
Experimental grouping and technical routes.

WT mice and 3 × Tg-AD mice (10 months old) were randomly divided into four groups: WT+Sham group, WT+OVX group, 3 × Tg-AD+Sham group and 3 × Tg-AD+OVX group. Each group consisted of 10 mice. Mice in the WT+OVX group and the 3 × Tg-AD+OVX group were injected intraperitoneally with 0.3% sodium pentobarbital for anesthesia and oophorectomy, and mice in the WT+Sham group and 3 × Tg-AD+Sham group underwent sham surgery to remove the adipose tissue around the ovary that was the same volume as the ovary. After completion of the surgery, each mouse required intraperitoneal injections of penicillin (20,000 units/per mouse) for three consecutive days. One month after completing the surgery, all mice underwent behavioral experiments to test their learning and memory abilities (Figure 1).

### 2.4 Behavioral tests

#### 2.4.1 Morris water maze (MWM)

MWM was an experiment used to evaluate the long-term spatial memory ability of mice, which was conducted for six days. For the first 1–5 days, a platform was hidden 1 cm underwater in the fourth quadrant and the mice were placed in the water from the first, second, third and fourth quadrants and allowed to move freely in the water for 60 s. If the mice did not find the platform hidden in the fourth quadrant within 60 s, the experimenter guided the mice to stay on the platform for 10 s; if the mice found the platform within 60 s, the time of finding the platform was recorded and the mice were allowed to stay on the platform for 10 s before the experimenter removed the mice from the platform. On the sixth day, the platform in the fourth quadrant was removed, the mice were put into the water from the second quadrant, and were allowed to move freely for 60 s before being fished out, and the number of times the mice crossed the platform within 60 s and the time they stayed in the fourth quadrant were recorded by using Topscan Package (Clever Sys, USA).

#### 2.4.2 Novel object recognition (NOR)

NOR was an experiment to assess the ability of mice to discriminate and learn, and the whole experiment was conducted for 3 days. On the first day, mice were placed in a square box and allowed to move freely for 5 min. On the second day, two cylinders of the same size and color were placed at the diagonal position of the box, followed by placing the mice in the box for 5 min of free movement. On the third day, a random cylinder (old object) was replaced by a square (new object) in the box, and then the mice were placed in the box for 5 min of free movement. The time spent exploring the new object and exploring the old object was recorded by using Topscan Package (Clever Sys, USA), and the recognition index of the mice was calculated [Recognition index = time spent exploring the new object/ (time spent exploring the new object + time spent exploring the old object)].

#### 2.4.3 Y-maze

The Y-maze was primarily used to assess the short-term learning and memory ability of mice. Mice were placed at the junction of the three sub-arms of the Y-maze and subsequently allowed to move freely for 10 min. The mice’s spontaneous alternation correct rate was recorded and calculated by using Topscan Package (Clever Sys, USA) [spontaneous alternation correct rate = number of correct arm entries / (total arm entries–2)].

#### 2.4.4 Elevated plus maze (EPM)

The EPM was used to assess anxiety-like behavior in mice. The EPM was 50 cm above the ground and consisted of 2 open arms and 2 closed arms, and the mice were placed at the junction of the 4 arms and allowed to move freely for 5 min, and the time spent in the open arms during the 5 min was recorded and analyzed using a Topscan Package (Clever Sys, USA) to determine the anxiety-like behavior of the mice.

#### 2.4.5 Forced swim test (FST)

The FST was used to assess depressive-like behavior in mice, in which mice were placed inside a hollow cylinder filled with water (a height of 45 cm and a radius of 10 cm) and allowed to move freely for 6 min, the first 2 min were not analyzed, and the immobility time of the mice during the last 4 min was analyzed using the Topscan Package (Clever Sys, USA) for assessing the depressive-like behavior of the mice.

### 2.5 Preparation of blood and brain tissues

After the behavioral experiments were completed, all mice were intraperitoneally injected with 0.3% sodium pentobarbital solution for anesthesia and cut off the mice’s beard, and the eyeball on the side of the mice was removed using forceps to take blood. After blood collection, mice were sacrificed by cervical dislocation. 3 mice were randomly selected in each group for cardiac perfusion, the mice chest cavity was opened, 10 ml of normal saline was injected from the left ventricle, followed by 10 ml of 4% neutral paraformaldehyde. After perfusion, the intact brain was isolated and stored in 4% neutral paraformaldehyde for 24 h, followed by paraffin embedding of the brain tissue and slicing into 5 μm thick sections for subsequent Nissl staining and immunofluorescence analysis. 6 mice in each group were randomly selected, and the brains were taken out and divided into 2 parts along the sagittal plane of the brain, selected the left brain stored in Golgi fixative and electron microscopy fixative, the Golgi fixative and the electron microscope fixative each preserved the left brain of 3 mice. The brain on the right was frozen with liquid nitrogen and stored in a refrigerator at −80°C. 6 right brains of mice and remaining mice brains were separated on ice, snap-frozen in liquid nitrogen and stored in a refrigerator at −80°C for subsequent physicochemical analysis.

### 2.6 Nissl staining

The sections were immersed in two vials of xylene solution for 15 min each, followed by immersion in graded ethanol (anhydrous ethanol, 95% ethanol, 90% ethanol, 80% ethanol, and 70% ethanol for 5 min each concentration), and transferred to distilled water for 2 min after 1 min of immersion in 70% ethanol. The sections were transferred to prepared toluidine blue solution for 5 min for staining and subsequently rinsed with distilled water. The sections were decolorized in 0.1% glacial acetic acid solution and transparent with xylene, and finally the sections were sealed with neutral resin and observed for Nissl body using an Eclipse microscope (Nikon).

### 2.7 Transmission electron microscopy

Tissues immersed in the electron microscope fixative were removed and fixed in 1% osmium acid (prepared in 0.1M phosphate buffer, pH = 7.4) for 2 h at room temperature, protected from light, and rinsed 3 times with 0.1M phosphate buffer for 15 min each time. The tissues were then dehydrated in gradient ethanol (sequentially in 30% ethanol, 50% ethanol, 70% ethanol, 80% ethanol, 95% ethanol, 100% ethanol, and 100% ethanol for 20 min each stage), followed by two immersions in 100% acetone for 15 min each. After sequentially embedded in a mixture consisting of acetone and EMBed 812 for 4 and 12 h, the tissues were subsequently placed in a 37°C oven overnight and placed in a 60°C oven for 48 h for polymerization. The tissues were cut into sections with a thickness of 60–80 nm using an ultrathin slicer (Leica, Leica UC7). Sections were stained in 2% uranyl acetate saturated alcohol solution protected from light for 8 min; washed 3 times in 70% alcohol; and washed 3 times in ultrapure water. The sections were placed in 2.6% lead citrate solution to avoid carbon dioxide staining for 8 min; washed with ultrapure water for 3 times, dried at room temperature overnight, and then observed and analyzed under a transmission electron microscope (HITACHI, HT7800/HT7700).

### 2.8 Golgi-Cox staining

Tissue was removed from the Golgi fixative and placed in Golgi staining solution for 14 days (changing the Golgi stain solution every three days). The tissues were immersed in the tissue treatment solution for 1 h, then replaced with a new tissue treatment solution and stored for 3 days away from light. The tissues were removed and cut into sections of 60 μm thickness using a microtome, washed with ultrapure water and Golgi stained dropwise, and the results were analyzed using pannoramic scanner software.

### 2.9 Immunofluorescence staining

Sections were sequentially placed in three vials of xylene solution for 15 min, followed by sequential immersion in graded ethanol (sequentially placed in anhydrous ethanol, anhydrous ethanol, 95% ethanol, 90% ethanol, 80% ethanol, 75% ethanol, and 70% ethanol for 10 min) and rinsed for 5 min with distilled water. Sections were heated and repaired by placing them in antigen repair solution for 20 min, and washed three times using PBS for 5 min each time after the sections cooled naturally. The samples were blocked with 3% bovine serum albumin for 30 min at room temperature, the blocking solution was discarded, and the corresponding antibody (ER-α, ER-β, thioflavine S, NMDAR2B, EAAT2, GABAA and GABAB) was added and incubated overnight at 4°C. The samples were washed 3 times with PBS for 5 min each time, and the corresponding secondary antibody was added and incubated for 1 h at room temperature away from light. After 3 washes with PBS, the sections were incubated with DAPI for 10 min at room temperature, followed by another 3 washes using PBS for 5 min each. Then, the sections were incubated in autofluorescence quencher for 5 min, washed in PBS and placed under a fluorescence microscope to be processed using Image Pro Plus software.

### 2.10 ELISA

The expression of E2, APP, tau, p-tau, Glu and GABA were detected using ELISA kits. Samples were pretreated according to the instructions that came with the ELISA kit, after which the kit was equilibrated at room temperature for 60 min and the 96-well/48-well plate was removed and set aside. Standards were added to the standard wells in ascending order of concentration, 50 μL per well, and 50 μL of the pre-treated sample solution was added to the sample wells. Subsequently, 100 μL of HRP-labeled detection antibody was added to each of the standard and sample wells, and a sealing mold was attached to the reaction wells and incubated at 37°C for 1 h in a water bath protected from light. After the incubation was completed, the waste solution was quickly discarded and 350 μL of washing solution was added to each well, which was allowed to stand for 1 min and then the washing solution was discarded, and the process was repeated 5 times. 100 μL of a mixture of Substrate A and Substrate B was added to each well and incubate at 37°C for 15 min in a water bath protected from light. After 15 min, 50 μL of termination solution was immediately added to each well protected from light. The absorbance value at wavelength 450 nm was detected using an enzyme marker within 15 min.

### 2.11 Western blotting (WB)

Brain tissue was mixed with pre-cooled protein lysate and thoroughly ground and centrifuged, and the upper layer of liquid in the centrifuge tube was aspirated and analyzed for protein quantification using the BCA kit. Subsequently, buffer was added and heated at 100°C for 20 min to ensure adequate protein denaturation. Proteins of different molecular weights were separated using 8–12% sodium dodecyl sulfate-polyacrylamide gel (SDS/PAGE) gels and the proteins were transferred using PVDF membranes. The PVDF membranes carrying proteins of different molecular weights were incubated with a blocking solution for 4 h at room temperature, followed by discarding the blocking solution and adding the corresponding primary antibody (ER-α, ER-β, PS1, Bcl-2, Bax, PSD95, AMPAR, NMDAR2A, NMDAR2B, EAAT1, EAAT2 and GABAB) for overnight incubation at 4°C, and then the membranes were washed with TBST for 4 times, each time for 5 min. The HRP-labeled secondary antibody was added and incubated at room temperature for 1 h, followed by 4 5-min washes of the membrane with TBST. Finally, the bands were visualized by using enhanced chemiluminescence kit, and were scanned using an AI-600 System (GE, USA). The gray values of the bands were quantitatively analyzed using Image J software.

### 2.12 Statistical analysis

All data were analyzed using GraphPad Prism version 8 (GraphPad Software, San Diego, CA, USA), and the experimental results were expressed as mean ± standard error (mean ± SEM). The normality of data was verified using the Shapiro–Wilk test, and their homogeneity of variance was verified using the Brown-Forsythe test. Differences among multiple groups were assessed for significance using 2-way (genotype and surgical intervention) ANOVA followed by *post hoc* Tukey tests, except for differences on days 1–5 in MWM, which were assessed using repeated-measures 2-way ANOVA. Differences associated with *P* < 0.05 were regarded as statistically significant.

## 3 Results

### 3.1 Effect of OVX on learning and memory abilities in 10-month-old 3 × Tg-AD mice

**FIGURE 2:**
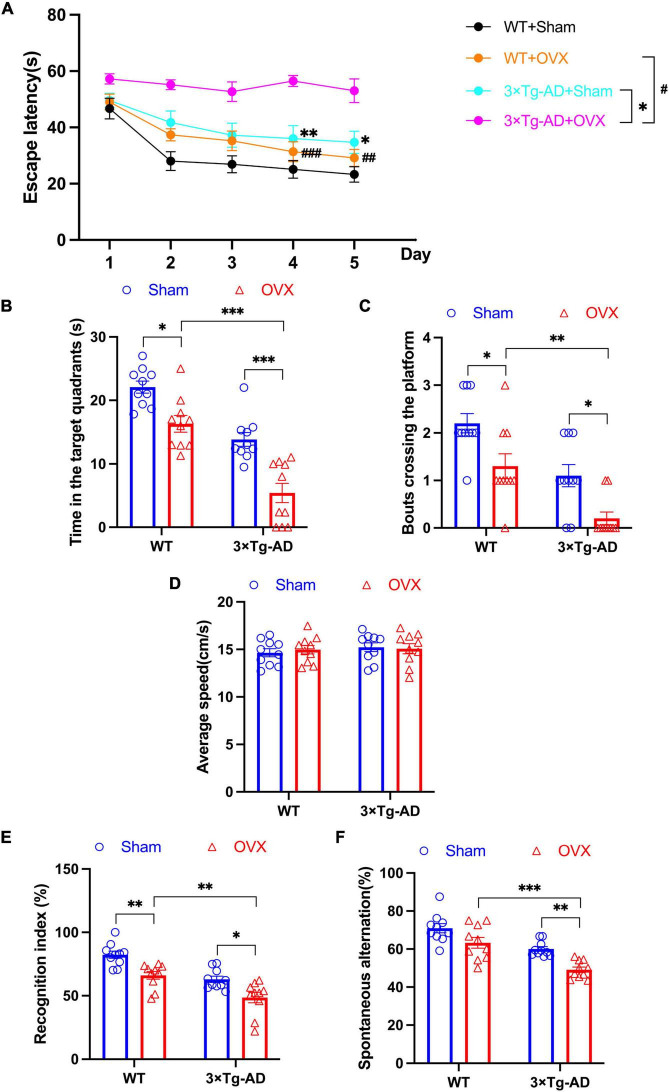
OVX impaired learning and memory abilities in mice. **(A)** Changes in the escape latency of mice on days 1–5 in MWM. **(B)** The length of stay in the target quadrant of mice on day 6 in MWM. **(C)** The number of crossings of the platform of mice on day 6 in MWM. **(D)** Average speed of swimming of mice on day 6 in MWM. **(E)** The recognition index of mice in NOR. **(F)** The percentage of spontaneous alternations of mice in Y-maze. Above all data are showed as means ± SEM; *n* = 10.^ ##^*P* < 0.01, ^###^*P* < 0.001; *P < 0.05, **P < 0.01, ***P < 0.001. The meaning of the symbol “#” is to compare the WT+OVX group with the 3 x Tg-AD+OVX group.

We performed MWM, NOR and Y-maze experiments to assess learning and memory abilities in 10-month-old 3 × Tg-AD mice. In MWM experiments, we assessed the escape latency of mice on days 1–5 and the length of stay in the target quadrant and the number of times they crossed the platform on day 6. We found that OVX increased the escape latency of 3 × Tg-AD+OVX mice on days 1–5 compared to 3 × Tg-AD+Sham mice (Day 4: *P* = 0.0068, Day 5: *P* = 0.0249; Figure 2A), we also observed a reduction in the residence time of the 3 × Tg-AD+OVX mice in the target quadrant and a decrease in the number of times they crossed the platform on day 6 compared to 3 × Tg-AD+Sham mice (*P* < 0.001 and *P* = 0.0241; Figures 2B, C). And OVX also led to the reduction of the above indicators of MWM in WT mice (*P* = 0.0111 for Figure 2B and *P* = 0.0241 for Figure 2C). In addition, we found that OVX caused much greater memory impairment in 3 × Tg-AD mice than in WT mice (*P* < 0.001 or *P* = 0.0013 for Figure 2A, *P* < 0.001 for Figure 2B and *P* = 0.0042 for Figure 2C). There was no significant difference in swimming speed between groups of mice (Figure 2D), suggesting that OVX does not affect mouse locomotion.

In addition, we evaluated the recognition index of mice in NOR experiments and the percentage of spontaneous alternation in Y-maze experiments. We found that both the recognition index and the percentage of spontaneous alternation were reduced in 3 × Tg-AD+OVX mice compared to 3 × Tg-AD+Sham mice (*P* = 0.0134 and *P* = 0.0038; Figures 2E, F). And OVX also led to the reduction of recognition index and the percentage of spontaneous in WT mice (*P* = 0.0046 for Figure 2E). In addition, we found that OVX caused much greater spatial memory impairment in 3 × Tg-AD mice than in WT mice (*P* = 0.0022 for Figure 2E and *P* < 0.001 for Figure 2F).

### 3.2 Effect of OVX on anxiety and depression-like behaviors in 10-month-old 3 × Tg-AD mice

**FIGURE 3:**
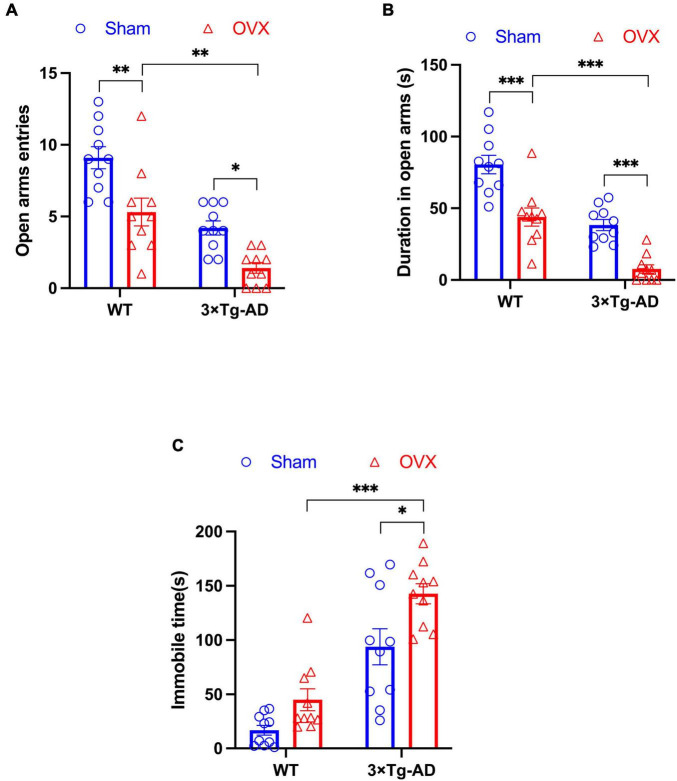
OVX caused anxiety and depression-like behaviors in mice. **(A)** The number of times mice entered open arms in EPM. **(B)** The residence time of mice in open arms in EPM. **(C)** The immobile time of mice in FST. Above all data are showed as means ± SEM; *n* = 10. *P < 0.05, **P < 0.01, ***P < 0.001.

We assessed changes in anxiety and depression-like behaviors in OVX mice by EPM and FST experiments. In EPM experiments, we found that 3 × Tg-AD+OVX mice had significantly reduced both number of entries and residence time in open arms compared to 3 × Tg-AD mice (*P* = 0.0328 and *P* < 0.001; Figures 3A, B). And OVX also led to the reduction the above indicators of EPM in WT mice (*P* = 0.0022 for Figure 3A and *P* < 0.001 for Figure 3B). Moreover, we found that after OVX, 3 × Tg-AD mice had more severe anxiety-like than WT mice (*P* = 0.0016 for Figure 3A and *P* < 0.001 for Figure 3B).

In addition, we observed an increase in immobility time in 3 × Tg-AD+OVX mice compared to 3 × Tg-AD mice in FST experiments (*P* = 0.0172; Figure 3C). And we found that after OVX, 3 × Tg-AD mice had more severe depression-like than WT mice (*P* < 0.001 for Figure 3C).

### 3.3 Effects of OVX on estrogen in the peripheral circulatory system and estrogen receptors in the brain of 10-month-old 3 × Tg-AD mice

**FIGURE 4:**
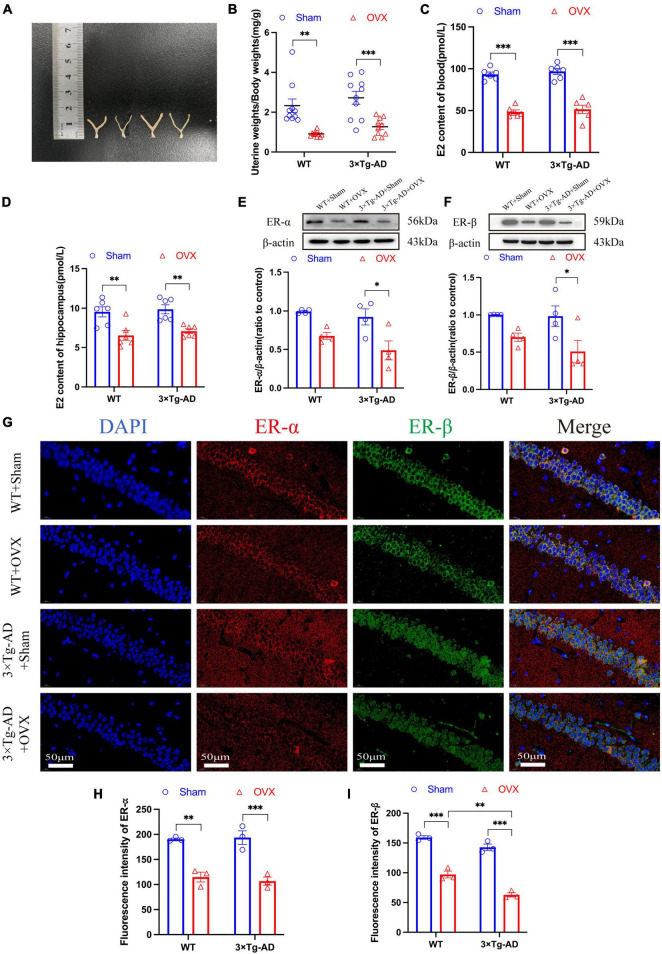
OVX reduced estrogen levels in peripheral blood and down-regulated the expression of ER-α and ER-β in the hippocampus of the brain in mice. **(A)** Pictures of mice uteruses, from left to right, WT+Sham, WT+OVX, 3 × Tg-AD and 3 × Tg-AD+OVX. **(B)** Quantitative analysis of mice uterine/body weights ratio. Data are shown as means ± SEM; *n* = 10. **(C)** Peripheral blood E2 levels in mice. Data are shown as means ± SEM; *n* = 6. **(D)** Hippocampus E2 levels in mice. Data are shown as means ± SEM; *n* = 6. **(E)** Expression of ER-α in the mice hippocampus. Data are shown as means ± SEM; *n* = 4. **(F)** Expression of ER-β in the mice hippocampus. Data are shown as means ± SEM; *n* = 4. **(G)** Representative immunofluorescence graphs of mice hippocampus for ER-α and ER-β (×600 magnifications; scale bar represents 50 μm). **(H)** Quantitative analysis of ER-α immunofluorescence intensity in the mice hippocampus. Data are shown as means ± SEM; *n* = 3. **(I)** Quantitative analysis of ER-β immunofluorescence intensity in the mice hippocampus. Data are shown as means ± SEM; *n* = 3. *P < 0.05, **P < 0.01, ***P < 0.001.

We compared the uterine/body weights ratio of OVX mice and mice without OVX, and we found that OVX caused uterine atrophy in mice (*P* = 0.0013 for WT or *P* = 0.0010 for 3 × Tg-AD; Figures 4A, B). In addition, we evaluated the effect of OVX on the estrogen-estrogen receptor pathway in mice by changing the expression of E2 in blood, E2 in hippocampus, ER-α and ER-β in mice, and our results showed that the E2 content in peripheral blood and hippocampus of OVX mice was significantly lower compared to mice without OVX (*P* < 0.001 for WT and 3 × Tg-AD, *P* = 0.0052 for WT and *P* = 0.0097 for 3 × Tg-AD; Figures 4C, D), and the WB results showed that OVX down-regulated the expression of ER-α and ER-β in the brain of 3 × Tg-AD+OVX mice compared to 3 × Tg-AD mice (*P* = 0.0153 and *P* = 0.0356; Figures 4E, F). And our immunofluorescence results also showed that OVX down-regulated the expression of ER-α and ER-β in mice (Figures 4G–I).

### 3.4 Effect of OVX on changes in pathological markers of AD in the brain of 10-month-old 3 × Tg-AD mice

**FIGURE 5:**
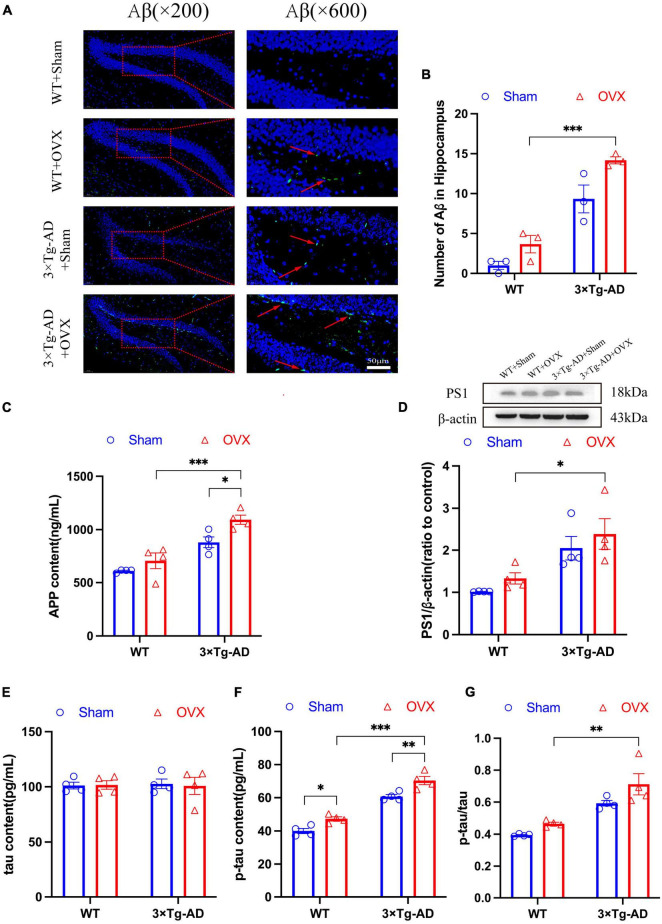
OVX resulted in up-regulated expression of Aβ, APP, and p-tau in the hippocampus of mice. **(A)** Representative micrographs by immunofluorescence for the Aβ in the hippocampal DG region (×600 magnifications; scale bar represents 50 μm). **(B)** Quantitative analysis of Aβ in **(A)**. Data are showed as means ± SEM; *n* = 3. **(C)** Concentration of APP in hippocampus. Data are showed as means ± SEM; *n* = 4. **(D)** Representative graphs by WB and quantitation of PS1. Data are showed as means ± SEM; *n* = 4. **(E)** Concentration of tau in hippocampus. Data are showed as means ± SEM; *n* = 4. **(F)** Concentration of p-tau in hippocampus. Data are showed as means ± SEM; *n* = 4. **(G)** The ratio of p-tau to tau. Data are showed as means ± SEM; *n* = 4. *P < 0.05, **P < 0.01, ***P < 0.001.

Impaired deposition and clearance of Aβ and hyperphosphorylation of tau proteins are the main pathological features of AD, so does OVX promote the expression of the above pathologic markers? Because the hippocampus of the brain is the main area responsible for memory storage, and the hippocampus is one of the first damaged areas in the brain of AD patients (Fjell et al., 2014). therefore, we detected the expression of AD pathological markers in the brains of OVX mice. We observed the number of Aβ plaques in the hippocampus of mice by immunofluorescence technology, and we found that the number of Aβ plaques in the hippocampus of 3 × Tg-AD+OVX mice increased compared to 3 × Tg-AD mice (Figures 5A, B). Furthermore, we found that after OVX, the number of Aβ plaques in brain of 3 × Tg-AD mice much more than WT mice (*P* < 0.001 for Figure 5B). We also measured the expression of APP and PS1, we found that both APP and PS1 expression were up-regulated in 3 × Tg-AD+OVX mice compared to 3 × Tg-AD mice (*P* = 0.0460; Figures 5C, D). In addition, we also measured the expression of tau in the brain of mice, and we found that the expression of total tau did not change in all groups of mice (Figure 5E), and the expression of p-tau was up-regulated (*P* = 0.0460 for WT or *P* = 0.0083 for 3 × Tg-AD; Figure 5F), the ratio of p-tau to tau was also increased in 3 × Tg-AD+OVX mice compared to 3 × Tg-AD mice (Figure 5G). In addition, we found that after OVX, the expression of APP, PS1, and p-tau proteins in the brains of 3 × Tg-AD mice were all higher compared with WT mice (*P* = 0.0390 for Figure 5D, *P* < 0.001 for Figures 5C, F).

### 3.5 Effect of OVX on apoptosis in the brain of 10-month-old 3 × Tg-AD mice

**FIGURE 6:**
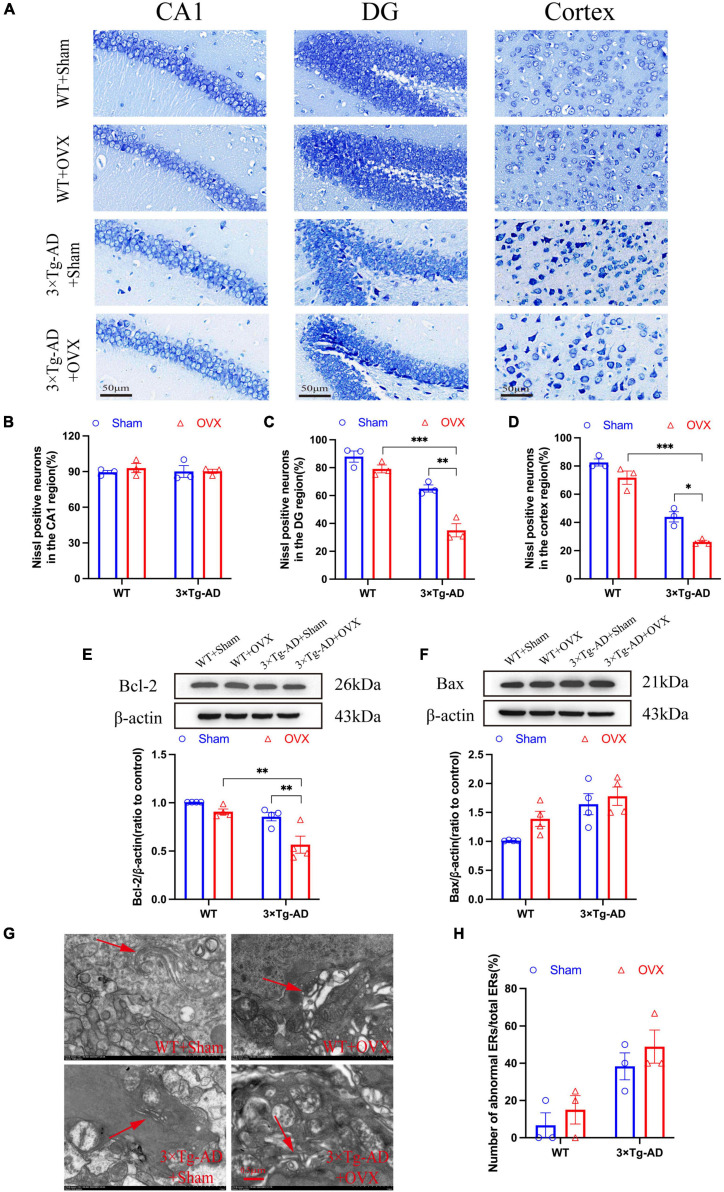
OVX promoted apoptosis and induces an ER stress in mice brain. **(A)** Representative micrographs of Nissl staining (×600 magnifications; scale bar represents 50 μm). Data are showed as means ± SEM; *n* = 3. **(B–D)** Quantitative analysis of Nissl positive neurons in the CA1, DG and cortex regions in **(A)**. Data are showed as means ± SEM; *n* = 3. **(E)** Representative images by WB and quantitation of Bcl-2. Data are showed as means ± SEM; *n* = 4. **(F)** Representative images by WB and quantitation of Bax. Data are showed as means ± SEM; *n* = 4. **(G)** Representative micrographs of ER in mice hippocampus (×15.0k magnifications; scale bar represents 0.5 μm). **(H)** Quantitative analysis of abnormal ERs and total ERs in **(G)**. Data are showed as means ± SEM; *n* = 3. *P < 0.05, **P < 0.01, **P < 0.001.

Excessive cell apoptosis was another major feature of AD and other neurodegenerative diseases. We detected cell apoptosis in the hippocampus and cortex of mice brain, the number of apoptosis in the hippocampus DG and cortex of 3 × Tg-AD+OVX mice was increased compared to 3 × Tg-AD mice (*P* = 0.0015 and *P* = 0.0205; Figures 6A, C, D). However, the apoptosis of the hippocampal CA1 region did not change significantly in all groups of mice (Figures 6A, B). We also measured the expression of Bcl-2 and Bax in the hippocampus of mice, and we found that the expression of Bcl-2 was down-regulated and Bax expression was up-regulated of 3 × Tg-AD+OVX mice compared to 3 × Tg-AD mice (*P* = 0.0080 for 3 × Tg-AD; Figures 6E, F). The accumulation of misfolded proteins in the brain would lead to endoplasmic reticulum (ER) dysfunction, which in turn would cause ER stress and promote apoptosis, so we observed the structure of ER by scanning electron microscopy, and we found that the ER in the brain of 3 × Tg-AD-OVX mice was proliferated, hypertrophied, and atrophied and disintegrated compared to 3 × Tg-AD mice (Figures 6G, H). In addition, after OVX, apoptosis was more severe in the DG and cortical regions of the brain in 3 × Tg-AD mice than in WT mice (*P* < 0.001 for Figures 6A, C, D).

### 3.6 Effect of OVX on the dendritic structure of neurons in the brain of 10-month-old 3 × Tg-AD mice

**FIGURE 7:**
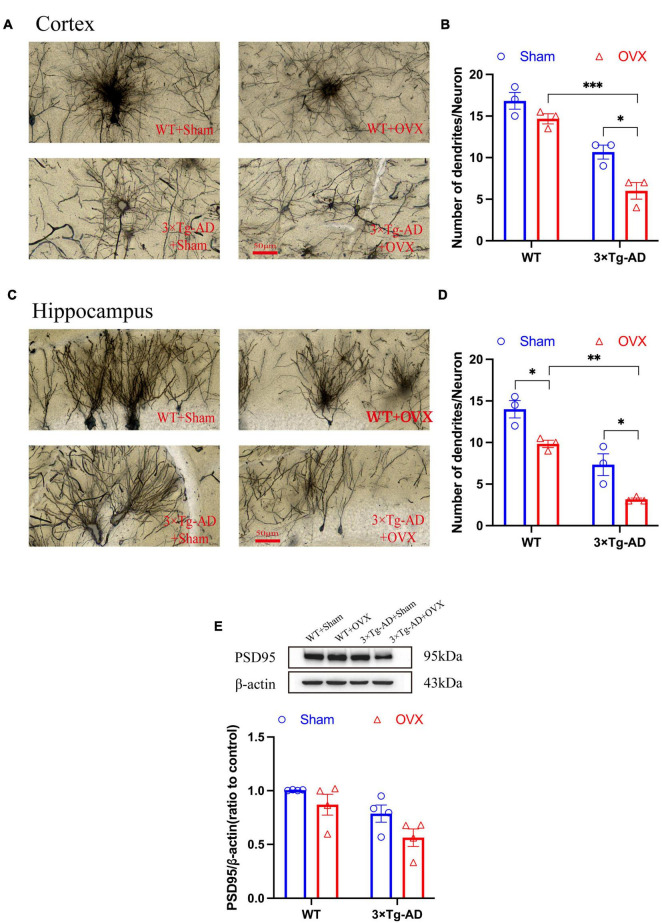
OVX damaged neuronal structures and down-regulated expression of PSD95. **(A)** Dendrites were observed in cortex of the mice brain by Golgi staining (×400 magnifications; scale bar represents 50 μm). Data are showed as means ± SEM; *n* = 3. **(B)** Quantitative analysis of the number of dendrites in **(A)**. Data are showed as means ± SEM; *n* = 3. **(C)** Dendrites were observed in hippocampus of the mice brains by Golgi staining (×400 magnifications; scale bar represents 50 μm). Data are showed as means ± SEM; *n* = 3. **(D)** Quantitative analysis of the number of dendrites in **(C)**. Data are showed as means ± SEM; *n* = 3. **(E)** Representative graphs by WB and quantitation of PSD95. Data are showed as means ± SEM; *n* = 4. *P < 0.05, **P < 0.01, **P < 0.001.

The structural integrity of neurons ensures the continuity of synaptic signaling and the smooth extraction of memory stores. We examined the number of neuronal dendrites in the hippocampus and cortex of the mice brain and the expression of PSD95. We found a substantial reduction in the number of neuronal dendrites in the hippocampus and cortex of 3 × Tg-AD+OVX mice compared to 3 × Tg-AD mice (*P* = 0.0231 and *P* = 0.0378; Figures 7A–D). In addition, the expression of PSD95 in the hippocampus of 3 × Tg-AD+OVX mice was also down-regulated compared to 3 × Tg-AD mice (Figure 7E). Furthermore, we found that the number of neuronal dendrites in the cortex and hippocampus of 3 × Tg-AD mice was much less than that of WT mice after OVX (*P* < 0.001 for Figure 7B and *P* = 0.0027 for Figure 7D).

### 3.7 Effect of OVX on the glutamatergic nervous system in the brain of 10-month-old 3 × Tg-AD mice

**FIGURE 8:**
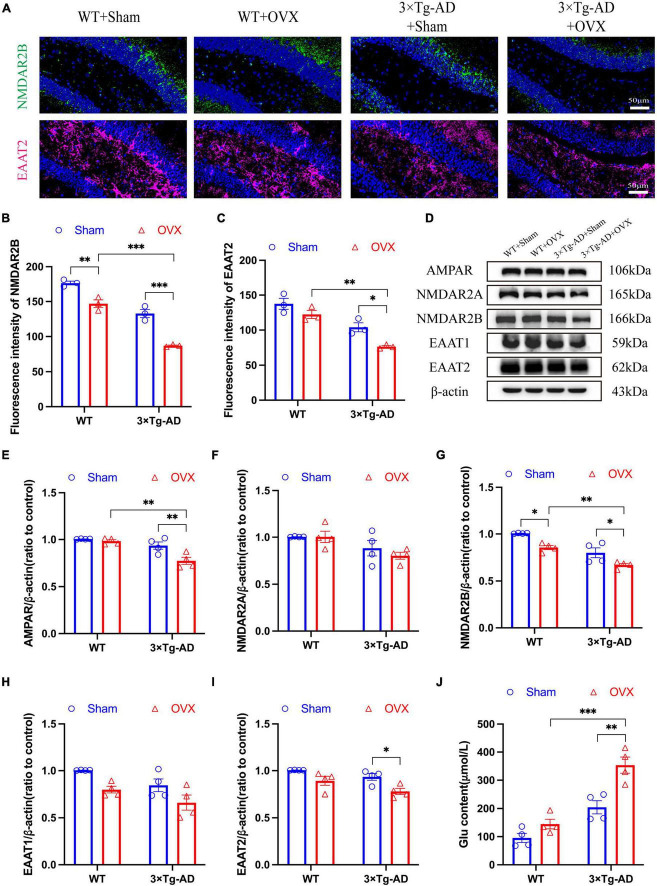
OVX caused disruption of the glutamatergic nervous system in the mice brain. **(A)** Representative micrographs by immunofluorescence for the NMDAR2B and EAAT2 in the hippocampal CA1 region (×600 magnifications; scale bar represents 50 μm). Data are showed as means ± SEM; *n* = 3. **(B)** Quantitative analysis of NMDAR2B in **(A)**. Data are showed as means ± SEM; *n* = 3. **(C)** Quantitative analysis of EAAT2 in **(A)**. Data are showed as means ± SEM; *n* = 3. **(D–I)** Representative images by WB and quantitation of AMPAR, NMDAR2A, NMDAR2B, EAAT1 and EAAT2. Data are showed as means ± SEM; *n* = 4. **(J)** Quantitative analysis of Glu in the mice hippocampus. Data are showed as means ± SEM; *n* = 4. *P < 0.05, **P < 0.01, ***P < 0.001.

Glutamatergic neurons were the most important excitatory neurons in the brain, and their overexcitation will lead to excitotoxicity. We evaluated the damage of OVX on neurons in the mice brain by detecting changes in Glu concentration, Glu receptors and Glu transporters. Our results showed that the expression of Glu receptors (AMPAR, NMDAR2A and NMDAR2B) in the hippocampus of 3 × Tg-AD+OVX mice was down-regulated to different degrees compared to 3 × Tg-AD mice (*P* < 0.001 for Figure 8B, *P* = 0.0072 for Figure 8E, *P* = 0.0428 for Figure 8G; Figures 8A, B, D–G) and Glu concentration of 3 × Tg-AD+OVX mice was increased (*P* = 0.0021; Figure 8J). Whereas in the brains of WT+OVX mice, only the expression of NMDAR2B was down-regulated (*P* = 0.0182; Figure 8G), and the expression of AMPAR and NMDAR2A was not significantly changed (Figures 8E, F). Subsequently, we examined the expression of EAAT1 and EAAT2, and we found that the expression of both EAAT1 and EAAT2 was down-regulated in the brain of 3 × Tg-AD+OVX mice compared to 3 × Tg-AD mice (*P* = 0.0332 for Figure 8J; Figures 8A, C, D, H, I). In addition, we found that after OVX, the expression of NMDAR2B protein in the brains of AD mice was lower than that of WT mice (*P* = 0.0042 for Figure 8G), and our immunofluorescence results confirm this finding (*P* < 0.001 for Figure 8B).

### 3.8 Effect of OVX on the GABAergic nervous system in the brain of 10-month-old 3 × Tg-AD mice

**FIGURE 9:**
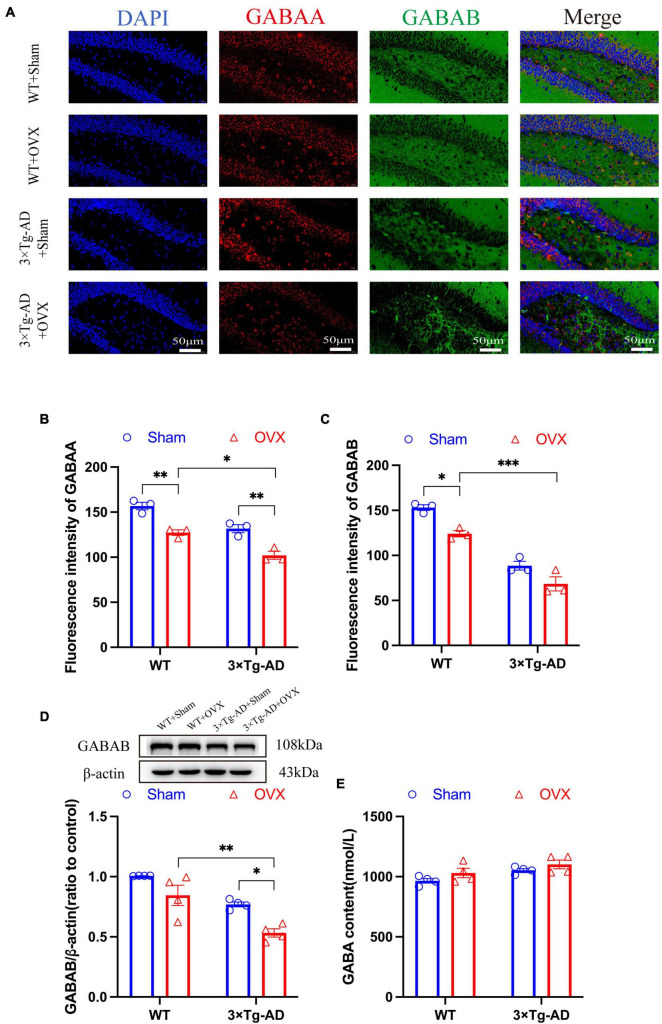
OVX damages the GABAergic nervous system in the mice brain. **(A)** Representative micrographs by immunofluorescence for the GABAA and GABAB in the hippocampal CA1 region (×600 magnifications; scale bar represents 50 μm). Data are showed as means ± SEM; *n* = 3. **(B)** Quantitative analysis of GABAA in **(A)**. Data are showed as means ± SEM; *n* = 3. **(C)** Quantitative analysis of GABAB in **(A)**. Data are showed as means ± SEM; *n* = 3. **(D)** Representative graphs by WB and quantitation of GABAB. Data are showed as means ± SEM; *n* = 4. **(E)** Quantitative analysis of GABA in the mice hippocampus. Data are showed as means ± SEM; *n* = 4. *P < 0.05, **P < 0.01, ***P < 0.001.

To evaluate the effect of OVX on the inhibitory nervous system in the excitation-inhibition balance system, we investigated the alterations of the GABAergic nervous system in OVX mice and detected the expression changes of GABA, GABAA, and GABAB. Our results showed that the expression of GABAA and GABAB in the brain of 3 × Tg-AD+OVX mice was significantly down-regulated compared to 3 × Tg-AD mice (*P* = 0.0039; Figures 9A–C), our WB results also confirmed that OVX led to down-regulation of GABAB expression in mice (*P* = 0.0166; Figure 9D). OVX also led to the down-regulation of expression of GABAA and GABAB in WT mice (*P* = 0.0040 for Figure 9B and *P* = 0.0169 for Figure 9C). In addition, GABA expression in the brains of 3 × Tg-AD+OVX mice was up-regulated compared to 3 × Tg-AD mice, but this trend of up-regulation was not very significant (Figure 9E), and we believed that this may be related to the small number of mice involved in the assay. In addition, we found that both GABAA and GABAB protein expression were differentially down-regulated in the brains of 3 × Tg-AD+OVX mice compared to WT+OVX mice (*P* = 0.0100 for Figure 9B, *P* < 0.001 for Figure 9C and *P* = 0.0166 for Figure 9D).

## 4 Discussion

In order to better mimic the pathological symptoms of clinical AD patients, researchers have developed a variety of transgenic mice. Among them, 3 × Tg-AD mice were able to overexpress APP, PS1 and p-tau proteins. Compared to other transgenic mice, 3 × Tg-AD exhibited multiple behavioral impairments at 10 months of age (Webster et al., 2014). Moreover, multiple neurotransmitter metabolic disorders were observed in their brains (Baek et al., 2023; Zhang M. et al., 2023). Importantly, 3 × Tg-AD female mice exhibit more prominent amyloid plaques, neurofibrillary tangle, and cognitive impairments than males (Yang et al., 2018). Estrogen was a sex hormone capable of influencing and regulating multiple systems in the human body and was mainly produced with the ovaries (Fuentes and Silveyra, 2019). Not only does it regulate reproductive development, but it was also important for the brain, bones, and blood vessels (Barth et al., 2023). Previous studies have indicated that estrogen deficiency led to cognitive impairment and anxiety-depression-like behaviors (Liu et al., 2019). Therefore, we combined OVX with 3 × Tg-AD mice to mimic the pathological development of postmenopausal AD patients. And our experimental results also confirmed that 3 × Tg-AD+OVX mice have more prominent cognitive impairment and anxiety-depression-like behaviors.

Senile plaques formed by extracellular Aβ aggregates and neurofibrillary tangles due to tau protein hyperphosphorylation were prevalent in the brains of AD patients and were considered to be the main pathological markers of AD development, and their synthesis and metabolism were regulated by multifactorial factors in the brain, in particular estrogen (Genazzani et al., 2007). Our findings showed that the expression of pathologic markers in the brains of 3 × Tg-AD mice was significantly upregulated after OVX, which exacerbated the AD condition. And this was also consistent with the changes in clinical postmenopausal AD patients (Mosconi et al., 2021; Depypere et al., 2023). In addition, the pathologic markers of AD in the brains of WT-OVX mice were also up-regulated to different degrees, suggesting that estrogen deficiency not only exacerbated the development of AD, but also promoted the development of AD.

*In vivo*, apoptosis and anti-apoptosis signaling were in a dynamic balance, and many diseases occurred due to the disruption of the dynamic balance of apoptosis signaling (Liu et al., 2013; Hu et al., 2021). Combined genetic downregulation and pharmacological blockade of the estrogen pathway increased apoptosis to varying degrees (Babiloni-Chust et al., 2022). Our results also showed that cells in the cortical and hippocampal DG regions of the brain of both WT+OVX and 3 × Tg-AD+OVX mice showed apoptosis to varying degrees. Bcl-2 and Bax were representative proteins of the anti-apoptosis and pro-apoptosis protein families, respectively. To further investigate the molecular mechanism of apoptosis caused by OVX, we examined the expression of Bcl-2 and Bax, and we found that the expression of the above two proteins was dysregulated in the brains of 10-month-old 3 × Tg-AD+OVX mice, which was consistent with previous studies (Sharma and Mehra, 2008; Sales et al., 2010), In addition, we found rupture and apoptosis in the endoplasmic reticulum in the brain of 3 × Tg-AD+OVX mice, suggesting that estrogen regulation of apoptosis is multifaceted (Meng et al., 2023).

The structural integrity of synapses was the guarantee of information reception and transmission between neurons, and the normal number and function of cytosolic dendrites of neurons were the basic requirements for the structural integrity of synapses. Neurological disorders, such as AD and depression, were associated with the loss of synapses in the frontal cortex and hippocampus (Holmes et al., 2019; Zhang J. et al., 2023), which may be closely related to estrogens, as in the brains of young or old monkeys with OVX, researchers have observed a significant reduction in the density of dendritic spines (Bailey et al., 2011), as well as some reduction in the expression of postsynaptic and presynaptic markers in the brain (Li et al., 2004). In contrast, our study found that the number of neuronal dendrites and the expression of PSD95 in 10-month-old 3 × Tg-AD+OVX mice were reduced to varying degrees, which suggests that estrogen deficiency has a certain degree of impairment on neuronal function and structure.

The hippocampus has long been recognized as an important region of the brain for processing long-term learning and memory (Bartsch and Wulff, 2015), and the basis for normal neurological function in the hippocampus relied on the dynamic balance of excitation-inhibition neurotransmitters (Ren et al., 2024). Glu and GABA were the most representative excitation and inhibition neurotransmitters in the brain, Elevated levels of Glu and GABA have been observed in the brains of postmenopausal AD patients (Liang et al., 2002; Jo et al., 2014). This was confirmed by our experimental results, and we also observed the occurrence of anxiety-depressive-like behaviors in 10-month-old 3 × Tg-AD mice induced by dysregulated neurotransmitter concentrations. Neuroprotection could be induced by direct estrogen-neuron interactions or indirectly mediated (such as estrogen-glial cell interactions) (Pawlak et al., 2005). But most of the previous studies on estrogen-neuroglia have been conducted in in young or non-transgenic mice (Taylor et al., 2010; Yun et al., 2018), and only single studies on glutamate or GABA and their receptors have been carried out (Theodosis et al., 2006; Yang et al., 2017; Nematipour et al., 2020). We chose 10-month-old 3 × Tg-AD+OVX mice for the first time in this experiment and studied glutamatergic and GABAergic neurons as a whole. Previous studies have pointed out that the expression of EAATs was affected by estrogen and Aβ (Lee et al., 2009; Tong et al., 2017), and we demonstrated that the expression of EAAT1 and EAAT2 was down-regulated in the brains of 10-month-old 3 × Tg-AD+OVX mice. Glu receptors and GABA receptors were receptors on the postsynaptic membrane that could bind specifically to Glu and GABA. Our results showed that the expression of both Glu and GABA receptors was downregulated to varying degrees, which we attributed to severe apoptosis or membrane receptor endocytosis induced by the superimposition of multiple factors (oxidative stress, inflammatory response, and Aβ deposition) due to OVX, which was a late stage in the progression of AD disease in 10-month-old mice (Wang et al., 2018; Martín-Belmonte et al., 2020; Alhowail and Aldubayan, 2023).

In summary, our findings suggested that estrogen deficiency induced by OVX worsens the cognitive impairments, and promotes apoptosis and damage to neuronal structure. Most importantly, estrogen deficiency disrupts glutamatergic and GABAergic neurons, resulting in the excitation-inhibition balance disturbance in the brain, which ultimately contributed to the development of AD.

## Data Availability

The raw data supporting the conclusions of this article will be made available by the authors, without undue reservation.
